# Postcoital bleeding is a predictor for cervical dysplasia

**DOI:** 10.1371/journal.pone.0217396

**Published:** 2019-05-23

**Authors:** Omer Cohen, Edwardo Schejter, Regina Agizim, Ron Schonman, Gabby Chodick, Ami Fishman, Anat Hershko Klement

**Affiliations:** 1 Department of Obstetrics and Gynecology, Meir Medical Center, Kfar Saba, Israel; 2 Maccabi Health Services, Tel Aviv, Israel; 3 Sackler School of Medicine, Tel Aviv University, Tel Aviv, Israel; University of Vermont Larner College of Medicine, UNITED STATES

## Abstract

**Background:**

Postcoital bleeding (PCB) is a common gynecological symptom that may cause concern among both patients and physicians. Current literature is inconclusive regarding management recommendations.

**Objective:**

To identify risk-factors for dysplasia/cancer among patients presenting post-coital bleeding (PCB).

**Methods:**

Using large health maintenance organization (HMO) database, all women reporting PCB in 2012–2015 were identified. PCB patient records in a single colposcopy center were reviewed. Age, marital status, ethnicity, gravidity, parity, BMI, smoking, PAP smear result (within 1 year of PCB presentation), colposcopy and biopsy results were recorded. Cases were matched by age and socio-economic enumeration area to controls accessing primary care clinics for routine care.

**Results:**

Yearly incidence of PCB ranged from 400 to 900 per 100,000 women; highest among patients aged 26–30 years. Among the sample of 411 PCB cases with colposcopy, 201 (48.9%) had directed biopsy. Biopsy results included 68 cervicitis (33.8%), 61 koilocytosis/CIN 1/condyloma (30.3%), 44 normal tissue (21.9%), 25 cervical polyp (12.4%), 2 CIN 2/3 (1%) and 1 carcinoma (0.5%). Positive predictive value for koilocytosis/CIN 1 or higher pathology was 15.6% (64/411) and 0.7% for CIN 2 or higher grade pathology (3/411). In conditional logistic regression, multiparty was a protective factor: OR 0.39 (95% CI 0.22–0.88, P = 0.02), while pathological PAP smear was a related risk-factor: OR 3.3 (95% CI 1.31–8.35, P = 0.01). When compared to controls, PCB patients were significantly (P = 0.04) more likely to present CIN 1 or higher grade pathology (OR 1.82, 95% CI 1.02–3.33).

**Conclusions:**

Study results indicate that PCB may require colposcopy, especially for nulliparous women with an abnormal PAP smear.

## Introduction

Postcoital bleeding (PCB) is a common gynecological symptom that may cause concern among both patients and physicians. Its prevalence varies from 0.7%-9% among menstruating women [1–3]. PCB may reflect a benign condition such as infection, but can also indicate the presence of pre-malignant condition or cervical cancer [4]. Colposcopy has been suggested as the appropriate investigative tool for ruling out cervical cancer or other pre-malignant pathology; however, the literature is inconclusive regarding management recommendations. The single systematic review published on this topic recommended against routine colposcopy [5]. There is currently no consensus regarding when PCB requires further investigation and when women can precede with routine gynecological follow-up. One of the main reasons for the lack of consensus is the paucity of data involving the prevalence of PCB in the population and the incidence of cervical cancer among these patients [5]. Other reasons include variations in study design, statistical analysis, and study location [6–8]. Therefore, management of PCB varies among countries [4].

The objectives of the current study was to evaluate the prevalence of PCB in the primary care setting, to assess the positive predictive value of the symptom for cervical pathology and to identify risk-factors for cervical pathology among these patients.

## Materials and methods

The study was approved by the Assuta Hospital Ethics Review Board (approval number 25/16). Maccabi Health Services is a nationwide health maintenance organization with 2.1 million insured customers. The study was based on a query of the database identifying all non-pregnant women ages 18–50 recorded as having PCB from January 1, 2012 through December 31, 2015. The current practice guidelines in Israel recommend colposcopy for every case of PCB. PCB patient records were sampled from a single colposcopy center and were reviewed. This center was chosen because of its supervised protection of patients' records. All colposcopies were performed by a single practitioner with more than 20 years of experience. Conventional cytology and colposcopy-guided biopsy were performed on all PCB patients. Age, marital status, ethnic background, gravidity, parity, BMI, smoking status, address (as a socio-economic status indicator), most recent PAP smear result (within 1 year), colposcopy evaluation and biopsy results were recorded. Pap cytology was performed as liquid based and classified according to the 2001 Bethesda system [9]. Pap test results were classified as within normal range, infectious, reactive, squamous cell abnormalities (atypical squamous cells of undetermined significance (ASC-US), atypical squamous cells high grade lesion not excluded (ASC-H), low grade squamous intraepithelial lesion (LSIL), high grade squamous intraepithelial lesion (HSIL) or squamous cell carcinoma and glandular atypia). For the purpose of analysis, all Pap smears classified as ASC-US or higher grade were considered pathological.

Cases were individually matched by age and socio-economic enumeration area to controls attending the primary care clinic for routine Pap.

### Data analysis

All calculations were performed using IBM SPSS 23.0 (IBM SPSS Inc., Armonk, NY, IBM Corp.). Normally distributed data were analyzed using unpaired student t test. Chi-squared or Fisher's exact test was used for comparing rates and proportions. Logistic regression was performed for prediction of cervical pathology. Matching analysis was performed using the McNemar's test. All P-values were tested as two-tailed and considered significant at <0.05.

## Results

### PCB incidence per 100,000 women

**Fig 1:**
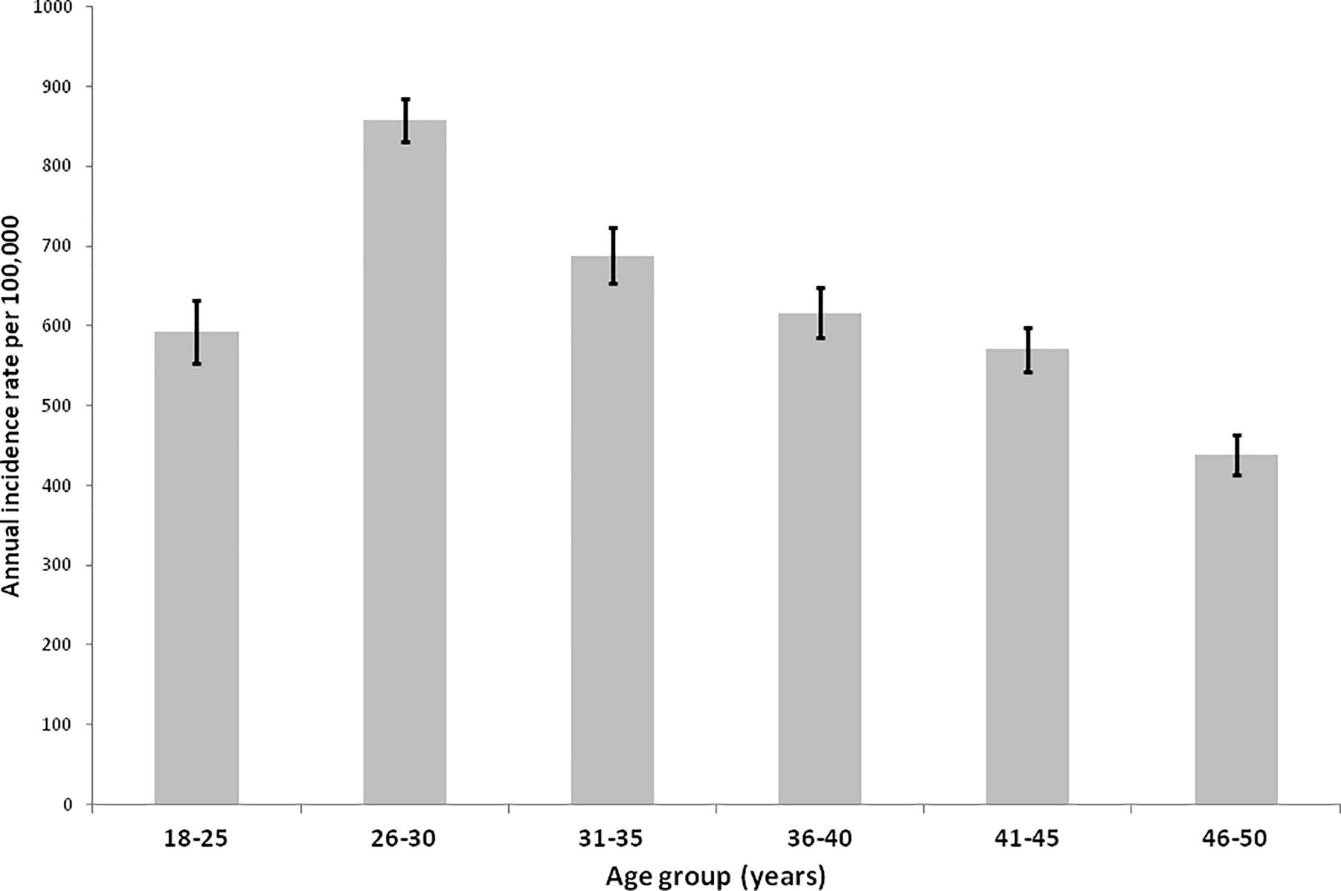
Incidence and 95% confidence intervals per 100,000 women presenting PCB, by age.

The incidence of PCB during the study period ranged from 400 to 900 annually per 100,000 women (Fig 1). The highest incidence was consistently observed among patients ages 26–30 years.

### Single center and case control analysis

A total of 411 consecutive PCB cases were reviewed. Demographic characteristics of the sample and subgroup analysis by past pap smear results are detailed in Table 1. A previous pathological Pap smear result in our PCB sample was significantly correlated with younger age, nulliparity and marital status (Table 1).

**Table 1:** Patient characteristics (mean ± SD or proportion (%)) by Pap smear result.

Characteristic	Total (N = 411)	Pathological Pap smear^1^ (N = 33)	Normal Pap smear (N = 372)
Age (years)	32±7.9	33.1±7.9*	29.5±6.3*
BMI (kg/m^2^)	24±4.3	24.1±4.3	22.8±3.9
Age at menarche (years)	12.9±1.3	13±1.3	12.8±1.3
Married	220/411 (53.7%)	N (52.4%)*	N (21.2%)*
Multiparous	202/409 (49.4%)	N (57%)*	N (15.2%)*
Smoker	70/408 (17.2%)	N (16.7%)	N (24.2%)

*P<0.05

^1^Defined as ASC-US or higher grade. Recent PAP smear was not available for 6 patients SD = standard deviation

A colposcopy-directed biopsy was performed in 201/411 cases (48.9%). All others were summarized as colposcopy without abnormal findings. Biopsy results were 68 cases of cervicitis (33.8%), 61 cases of koilocytosis/CIN 1/condyloma (30.3%), 44 cases of normal tissue (21.9%), 25 cases of cervical polyps (12.4%), 2 cases of CIN2/3 (1%) and one case of carcinoma (0.5%).

A significantly higher proportion of patients were not required to undergo biopsy during the colposcopy examination, in case the PAP smear was normal (53.5% versus 27.3%, P <0.01). The biopsy results were also distributed in a significantly different manner in case the PAP smear was normal compared to an abnormal smear: Cervicitis and benign polyps were more common among normal background PAP smears, while HPV-related pathologies were more common among the abnormal background PAP smear. The single carcinoma case in the current cohort was actually diagnosed in a patient with a normal background smear and other lower grade HPV related pathologies were present in 12.1% of patients with a recent normal PAP.

Once presenting with PCB, positive predictive value for a cervical pathology of koilocytosis/CIN 1 and higher was therefore 15.6% (64/411) and 0.7% for high grade pathology of CIN 2 or higher (3/411). Conditional logistic regression analysis for CIN 1 or higher, found that marital status, age at bleeding, BMI and smoking were not correlated with pathology. Multiparty was found to be a protective factor: OR 0.39 (95% CI 0.22–0.88, P = 0.02), while pathological PAP smear was a significant risk factor: OR 3.3 (95% CI 1.31–8.35, P = 0.01).

Sufficient matching was available for 259 cases. We studied the records of the controls, tracked all those who required a colposcopy and reviewed the colposcopic reports. When compared to controls, PCB patients were significantly (P = 0.04) more likely to present CIN 1 or higher grade pathology (OR 1.82, 95% CI 1.02–3.33).

## Discussion

This study supported PCB as a common complaint in the primary care setting among women of reproductive age. The presence of PCB was correlated with cervical pathology; the risk was twice that of the background population. Among patients presenting with PCB, the probability of cervical pathology was independently related to nulliparity and to Pap smear classified as ASC-US or higher.

The incidence of PCB in our population is higher than that recently reported; It was 39-59/100,000 among Taiwanese women, where 2.3% of patients reporting PCB had a diagnosis of cancer [10]. It is probable that the high availability of health care services, including community-based colposcopies in our setting and the national health insurance coverage can explain the relatively-high documented frequency of this symptom. In Britain, the self-reported mean annual cumulative incidence was very high, reaching 6% in menstruating women [2]. However, as the figure was based on self reports, it is difficult to compare it to our population. If we extrapolate our calculated incidence to approximately 30 years of reproductive age span (18–50 years), the resulting prevalence would be approximately 1%. A 1% prevalence is within the range reported by previous groups [3, 4]. The prevalence of PCB is obviously much higher in the tertiary care setting: in a retrospective report from Turkey published in 2015[11], the PCB prevalence was 15.9% among patients referred to the center. All patients studied were already diagnosed with either an abnormal apperaing cervix during a routine follow-up or an abnormal finding during the routine screening process [11].

The management of PCB is not uniform, referral criteria for follow-up differ and therefore, diverse baseline pathology rates are reported. In the UK for example, primary care was given by a general practitioner (GP): among 137 women referred to a tertiary center, 1 was reported to have carcinoma, for a rate of 0.7% [12]. Some of these patients were urgently referred. The authors describe that colposcopy was performed for only 46% of referred patients, even in the hospital setting, due to shortage of resources [12]. This finding of 0.7% is lower than that in an earlier report from the UK [13], where a 4% rate of invasive cervical cancer was reported among 314 women presenting with PCB. Among Taiwanese women, 2.3% of patients reporting PCB were diagnosed with cervical cancer and women with PCB had a 1.47-fold risk of cervical dysplasia and 1.59-fold risk for malignant neoplasm of cervix [10]. In a systematic review [5] published in 2006, the predictive value of PCB for cervical cancer was defined as poor; researchers concluded that due to the high incidence of PCB and high cost of colposcopy, it would appear to be inappropriate to investigate all women presenting with this symptom for cervical cancer. Drawing conclusions was however intriguing due to variations of both presenting symptoms and management: Additional studies on the epidemiology and gynecological malignancy in the community and primary care were required. Since 2006, additional studies were initiated to address PCB management. In a prospective study published in 2010, colposcopy was recommended for PCB lasting more than 4 weeks [12]. A questionnaire-based study among perimenopausal women ages 40–54 years [14] found 51% rate of spontaneous resolution without recurrence for 2 years. Of the 785 women identified with intermenstrual and/or PCB, only one developed uterine cancer. Again, authors stated that the association of PCB symptoms with malignancy is weak [14]. Some groups tried to refine the predictive value of PCB by adding Pap smear results to the management protocol [15] to increase the specificity. In a study from 2006, among 142 women evaluated with colposcopy, the risk of CIN in a woman with PCB and abnormal cervical smear were more than two-fold, as compared with a negative smear (odds of 0.47 and 0.19, respectively, with a relative risk (RR) of 2.37) [15]. In a study from 2007, 87 women with PCB and negative cytology underwent colposcopy; none was diagnosed with cancer and 3.5% were CIN 2/3. Therefore, this study was supportive of much greater risk of cervical neoplasia than in the general population, even with a normal Pap smear [16]. In 2015, a Turkish group studied the presence of PCB complaint among patients referred to a tertiary center due to positive cervical screening findings or due to a concern raised by the primary care physician during the cervical inspection[11]. They report PCB presence in 237 out of 1491 patients referred. They did not find PCB as a significant risk factor for CIN 2 (analysis was directed solely to CIN 2 pathology), but the setting and the studied denominator are critically different from our primary care population.

In the current study we were able to provide symptom incidence, based on a population survey and to study a sample that is relatively large as compared to previous reports. Though our sample is limited, we were also able to assure uniformity of assessment, since current health policy in Israel dictates colposcopy for every case of PCB[17]and all colposcopies were performed by a single, highly experienced practitioner. By matching patients according to socioeconomic status, we could efficiently assess the odds for cervical HPV pathology as compared to a routinely screened population.

Previous works, as well as ours, generally support PCB as a significant risk factor for cervical dysplasia. Appropriate management should be conducted according to medical resources and the prevalence of cervical dysplasia/cervical malignancy in the specific population. Other risk factors for cervical pathology and symptom persistence should be considered as well.

To conclude, according to our study, PCB may require colposcopy, especially for nulliparous women with an abnormal PAP smear.

## Supporting information

S1 Dataset (SAV)
